# Research on Polysaccharide–Protein Composite Hydrogels for Gastrointestinal Targeted Delivery: A Review

**DOI:** 10.3390/gels12020168

**Published:** 2026-02-14

**Authors:** Jingjing Guo, Yuxin Cai, Ran Zou, Chen Ai, Qun Fu

**Affiliations:** 1 Key Laboratory of Forest Resources Utilization of Heilongjiang Province, College of Food and Health, Northeast Forestry University, Harbin 150040, China; guojingjing@nefu.edu.cn (J.G.); 2023123110@nefu.edu.cn (Y.C.); 15946087132@163.com (R.Z.); 2 Scotland’s Rural College, The University of Edinburgh, King’s Building, West Mains Road, Edinburgh EH9 3JG, UK

**Keywords:** polysaccharide–protein composite hydrogels, gastrointestinal tract, targeted delivery, mechanism

## Abstract

Polysaccharide–protein composite hydrogels have demonstrated remarkable potential in targeted gastrointestinal delivery owing to their excellent biocompatibility, adjustable physicochemical characteristics, and intelligent responsiveness. This review provides a comprehensive overview of the underlying mechanisms and diverse applications of these composite hydrogels in gastrointestinal targeted delivery, with a particular emphasis on their stimuli-responsive release behaviors triggered by internal and external factors such as pH, enzymes, magnetic fields. Special attention is also given to their advantages in protecting sensitive bioactive ingredients, including curcumin, EGCG, probiotics. Furthermore, this review highlights their capabilities in achieving high encapsulation efficiency, smart controlled release and targeted delivery, while also presenting current challenges associated with material stability, targeting precision, large-scale production, and clinical translation. Finally, future perspectives are discussed, focusing on the development of multi-response system design, innovative biomaterials, advanced manufacturing technology applications, and AI-assisted optimization. These directions aim to provide theoretical foundations and technical strategies for advanced research and practical applications of polysaccharide–protein composite hydrogels in a targeted gastrointestinal delivery system. Overall, this review underscores the significant promise of polysaccharide–protein composite hydrogels as intelligent gastrointestinal delivery platforms and provides a systematic reference for their rational design and future translational development.

## 1. Introduction

Gastrointestinal targeted delivery represents a key strategy in modern drug development aimed at improving the efficacy of oral administration in complex gastrointestinal environments. By enabling site-specific drug release, this approach offers the potential to enhance therapeutic performance, reduce systemic side effects, and support innovation in pharmaceutical formulation.

The gastrointestinal tract imposes multiple physiological and biochemical barriers to effective drug delivery. Gastric acidity and digestive enzymes can degrade many therapeutic agents, while the intestinal mucus layer and epithelial tight junctions further restrict absorption [1]. These combined factors markedly limit the effectiveness of conventional oral delivery, particularly for macromolecular drugs and labile bioactive compounds. To address these challenges, intelligent targeted delivery systems have been developed to respond to pathological microenvironmental cues, such as pH gradients, enzyme activity, and redox conditions, thereby enabling localized and controlled drug release [2,3,4]. Such strategies have demonstrated improved local drug availability and therapeutic outcomes while enhancing patient compliance in chronic gastrointestinal diseases [3,4]. By 2031, the market sales of sustained-release dosage forms are projected to reach $1127 million [5].

**Figure 1:**
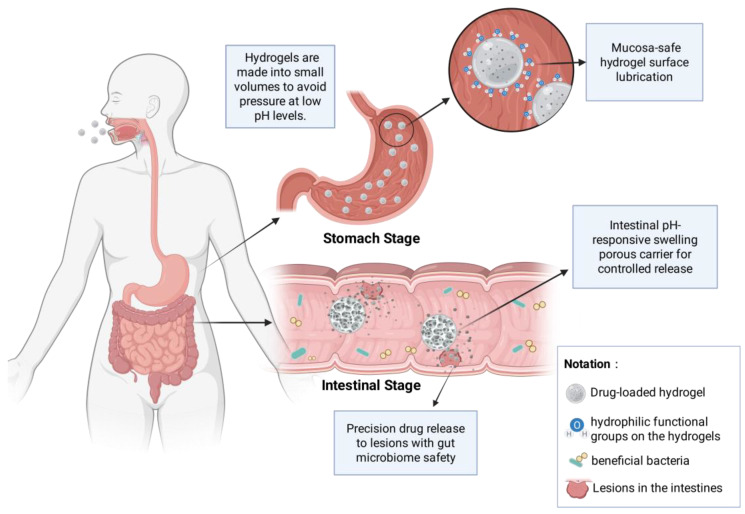
Gastrointestinal-Friendly Delivery of Smart Hydrogels: Schematic of the Biosafety Mechanism.

To overcome these barriers, delivery carriers with high environmental adaptability are urgently required. Hydrogels have attracted considerable attention for gastrointestinal drug delivery due to their three-dimensional network structures and responsiveness to pH and enzymatic variations along the gastrointestinal tract. These properties allow hydrogels to protect encapsulated bioactive substances under gastric conditions and subsequently enable controlled release in the intestinal environment [6,7,8,9], In addition, hydrogels constructed from physically crosslinked natural polymers, including chitosan, sodium alginate, and other polysaccharides, exhibit excellent biocompatibility and biological safety, minimizing chemical irritation and supporting oral administration [7,10,11]. As shown in Figure 1, the smart hydrogel drug delivery system adapts its structure in response to different gastrointestinal environments: it remains compact in the acidic stomach to minimize pressure and then swells into a soft, porous network in the neutral intestines for targeted drug release. Moreover, hydrogels exhibit remarkable functional scalability. Through modifying the surface of targeted ligands (e.g., thiolation modification [12]) or integrating with functional nanomaterials [11].

Among hydrogel-based systems, polysaccharide–protein composite hydrogels have emerged as particularly promising platforms for gastrointestinal targeted delivery. The synergistic integration of polysaccharide and protein components combines mechanical stability, environmental responsiveness, hydrophilicity, and bioadhesive properties, enabling structural integrity and functional stability under dynamic gastrointestinal conditions [13], Compared with single-component hydrogels, composite systems more effectively suppress premature drug release in gastric environments and allow programmable, multi-stage release profiles in the intestine [14,15,16], Furthermore, many polysaccharides possess intrinsic prebiotic activity, while protein components may provide nutritional or bioactive functions, enabling composite hydrogels to serve as integrated “therapy–nutrition” platforms for gastrointestinal disease management [11,17,18,19].

This review systematically searched databases, including Web of Science, PubMed, and Scopus, for studies published in the last three years on polysaccharide–protein composite hydrogels for gastrointestinal targeted delivery. Inclusion criteria comprised experimental research, studies involving polysaccharide–protein composite systems, explicit focus on gastrointestinal delivery mechanisms or applications, and publication in English. Exclusion criteria encompassed: non-gastrointestinal delivery applications and literature lacking experimental data or mechanism discussions.

## 2. Mechanism of Gastrointestinal Targeted Delivery by Polysaccharide–Protein Hydrogel Delivery Systems

Protein–polysaccharide hydrogels represent a paradigm shift in gastrointestinal targeting, moving beyond passive carriers to intelligent platforms that exploit specific physiological cues. By engineering materials like chitosan/pectin for precise pH-dependent erosion or designing konjac glucomannan systems for enzymatic degradation by colonic microbiota, these hydrogels achieve site-specific drug release with high temporal and spatial control. This strategic delivery, demonstrated to enhance intestinal probiotic colonization by over 250-fold [20] and minimize systemic exposure to drugs like 5-ASA [21], effectively mitigates off-target effects such as nephrotoxicity. The field is now advancing toward multi-stimuli responsive systems capable of navigating the GI tract’s complexities for the delivery of sensitive biomacromolecules.

### 2.1. Physiological Environment-Based Response Release

#### 2.1.1. pH-Responsive Release

**Figure 2:**
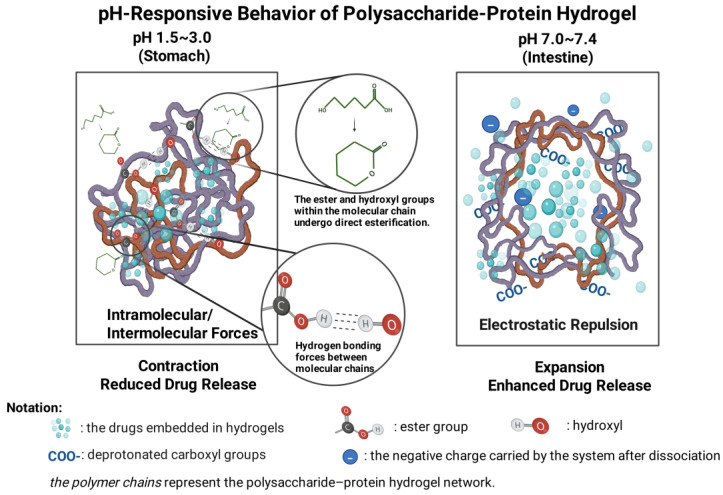
Diagram of the pH-responsive hydrogel mechanism of polysaccharides and proteins.

The gastrointestinal tract exhibits a heterogenic pH gradient (stomach pH 1.0–3.0, small intestine pH 6.0–7.0, colon pH 7.0–7.4). pH-responsive hydrogels are formed by a three-dimensional polymer network containing acidic or basic functional groups. Their charge density, hydrophilicity/hydrophobicity, and crosslinking stability vary with the change in pH. At different pH levels, the network structure of polysaccharide–protein hydrogels undergoes dynamical changes that primarily stem from the protonation or deprotonation of chain-bound ionizable groups (such as carboxyl and amino groups). At the low pH, -COOH and -OH groups can form lactone rings or intramolecular/intermolecular interactions. This reaction reduces the electrostatic repulsion and enhances the crosslinking and contraction, leading to decreased swelling capacity and restricted drug release [22,23]. As the pH increases, -COOH undergoes deprotonation to form COO^−^, generating electrostatic repulsion that causes the molecular chain to extend and the hydrogel to inflate, hence boosting drug release (Figure 2) [24,25]. For instance, whey protein–sodium alginate (WPI-SA) composite hydrogels which are made using the ion-gelation technique to transport flavonoids (TF) showed notable pH-responsive release properties [26]. The encapsulated TF is well protected by this hydrogel’s modest swelling rates in the acidic stomach environment (pH 2.0). It quickly expands and releases almost all of the TF in the neutral gut environment (pH 7.4), exhibiting outstanding pH reversibility and targeted delivery capacity.

Another way to achieve pH responsiveness is to create a highly biocompatible hydrogel by generating a three-dimensional network structure or stimulating the precursor material with a crosslinking agent at a certain pH [27]. For instance, through NH_3_^+^·COO^−^ ion interactions, the carboxymethyl chitosan–sodium carboxymethyl cellulose (CCS/CMC-Na) composite hydrogel spontaneously gels in stomach acid, creating a physical barrier to shield the active components. Hydrogel disintegration results from carboxylate protonation, which reduces hydrogen bonding and electrostatic repulsion in the neutral colonic environment [11]. Such phase-change activity extends the duration of drug release, preserves structural integrity, and improves the hydrogel’s responsiveness to physiological settings.

#### 2.1.2. Enzyme-Triggered Release

Apart from pH responsiveness, another essential technique for achieving tailored distribution is enzyme-triggered release. A diverse range of unique enzyme systems, including azoreductases, glycosidases, and polysaccharide-degrading enzymes, are produced by the colon’s abundant microbial life. These enzymes may precisely break down certain polysaccharide components, allowing for colon-specific release; however, they are less common or active in the stomach and small intestine. The enzyme-triggered drug release systems have many advantages over the selectivity, specificity, high efficiency, and the capacity to accelerate polymer breakdown under moderate circumstances [28]. For example, chitin can be broken down by Bacteroides’s chitinase [29], resistant starch and some cell wall-associated polysaccharides can be broken down by corresponding colonic enzymes [30], and guar gum can be broken down by extracellular colonic enzymes such as mannanase, α-galactosidase, and β-mannosidase.

Enzyme-triggered systems are commonly represented by β-Cyclodextrin (β-CD) and its derivatives. β-CD is a cyclic oligosaccharide made up of seven glucose units. Various drug molecules can be encapsulated in their hydrophobic cavity to create “host-guest” inclusion complexes, which shield medications as they transit through the upper digestive system. Enzymes generated by Bacteroides bacteria, unique to the colon, may break down β-CD, allowing for colon-targeted medication release, while human digestive enzymes cannot destroy it [12]. According to studies, the human diclofenac-β-CD conjugate has a delayed peak concentration (tmax) of ten hours. Fecal metabolic analysis in rats confirmed its degradation via Bacteroides-mediated action [31]. Inulin was used by Zhang et al. [32] to administer olsalazine (Olsa). Colonic inulinase particularly breaks down inulin, which not only guarantees the gel’s biocompatible metabolism but also alters the hydrogel network structure. Cu_2_(Olsa) nanonails are released gradually and continuously throughout the colon as a result, improving local medication concentration and lowering systemic adverse effects. Furthermore, the AP shell of the nanoparticles in EMNs-gel may be selectively hydrolyzed by matrix metalloproteinases (MMPs), which are overexpressed in ulcerative colitis lesions in terms of releasing Fe^3+^. This encourages the gel to reconstitute from a cohesive mass into a very sticky hydrogel, allowing for targeted hemostasis and anti-inflammatory treatment [33].

In addition to polysaccharide components, protein components can potentially contribute to enzyme responsiveness. However, because proteases (like trypsin) are present throughout the stomach, their breakdown by these enzymes lacks selectivity. A more popular tactic is conjugating proteins with certain polysaccharides to obtain more accurate colonic targeting, or using enzymes unique to the colonic microbiota to break down particular polysaccharides. The soy protein–soybean hull polysaccharide double-crosslinked liposome gel, for instance, makes use of two different enzymes’ capacities for breakdown [34]. Enzyme-responsive hydrogels have great prospect for treating gastrointestinal and systemic disorders by means of targeting drug delivery based on disease-specific enzymatic activity. The variety and diversity of the gut bacteria, however, might result in erratic release behavior, calling for the creation of more individualized and manageable hydrogel formulations [35].

Overall, physiological environment–responsive systems enable gastrointestinal targeting by exploiting endogenous cues such as pH gradients and microbial enzymes; however, pH-responsive hydrogels generally provide robust and predictable release at the segmental level, whereas enzyme-triggered systems offer higher site specificity at the cost of increased biological variability and reduced inter-individual consistency.

### 2.2. Regulation in Response to External Stimuli

#### 2.2.1. Magnetothermal Response

In recent years, medication delivery technologies have significantly progressed to reduce adverse pharmacological effects. These materials have garnered a lot of attention since magnetic-responsive hydrogels have external field-modulation capabilities, and human cells are immune to external magnetic fields [36,37,38]. It is possible to generate magnetic hydrogels by combining inorganic (such as iron oxide) and organic (such as polysaccharide) ingredients. By contrasting two techniques for creating chitosan hydrogels, Meisan Sadeghi et al. showed that formaldehyde crosslinking is more suited for loading FeO_4_ superparamagnetic nanoparticles. After being immobilized in the hydrogel, the resultant FeO_4_ nanoparticles (9.8 nm in diameter) maintained their superparamagnetic characteristics, which include directional alignment in magnetic fields. They simplified the separation method and increased the rate of bovine serum albumin (BSA) separation from 48% to 70% by utilizing magnetic response [39]. This work shows that the magnetic response qualities from FeO_4_-chitosan hydrogel composite system may solve the laborious and ineffective problems of conventional BSA separation techniques, and thus offer novel materials and technical strategies for magnetically assisted biomolecule separation.

Infection with Helicobacter pylori also presents serious health hazards. Targeted antibacterial activity and synergistic therapy can be accomplished by using the heat-generating capabilities of magnetic materials under alternating magnetic fields (AMF) as well as their magnetic field localization effects. In AMF, magnetic particles produce heat through rotating friction, and dissipative losses associated with Neel-Brownian relaxation raise local temperatures even further. This dual mechanism destroys the flagellar structures of helicobacter pylori and inhibits its cellular attachment [40,41]. A graphene-encapsulated iron-cobalt alloy (FeCo@G) magnetic heater was developed by Xia et al. [42] that, more significantly, increases the expression of heat shock protein 70 (HSP70) in gastric epithelial cells in addition to directly eliminating Helicobacter pylori by localized thermotherapy. HSP70 is an essential cytoprotective protein that prevents infection-induced pathophysiological damage, preserves mucosal integrity, and reduces stomach inflammation. Thus, one of the main goals in creating magnetoresponsive hydrogels is to encapsulate or link magnetic nanoparticles (such as FeO_4_, FeO_3_, and CoFeO_4_) within networks of polysaccharide–protein hydrogels [43].

Remote, non-invasive, and accurate control is made possible via magneto-thermal response methods. To improve local medication concentration and get around dispersion problems brought on by gastrointestinal motility in oral formulations, magnetic hydrogels can be guided by an external static magnetic field to travel throughout the gastrointestinal system and aggregate at the target spot. However, local heating over its lower critical solution temperature (LCST) causes the network to rapidly collapse, “expelling” the medication if the hydrogel contains thermosensitive components (such as gelatin or particular synthetic thermosensitive polymers). This is the main application for alternating magnetic fields. Heating increases drug diffusion rates to provide “on-demand” pulsed administration by speeding up molecular mobility and segmental relaxation, even in hydrogels that are not thermosensitive [37]. According to research, a dual pH-and magnetically sensitive oral delivery system may be created by encapsulating superparamagnetic iron oxide nanoparticles (SPIONs) in magnetic liposomes (MLPs) and by embedding them into alginate Pickering emulsion hydrogel beads. The system has a strong magnetothermal effect when stimulated by AMF, which efficiently produces localized heat and greatly increases the release rate of hydrophobic medications (like curcumin) in colonic and intestinal fluid simulations [44]. Based on fibrin and hyaluronic acid, Jorge et al. [45] created an ultra-soft hydrogel that is magnetically sensitive. They methodically examined its solvent release behavior under external magnetic fields using a mix of modeling and tests. There was a 67% increase in magnetically driven release compared to the non-magnetic control. Additionally, because the material showed more rigidity and less deformation at higher frequencies, low-frequency dynamic magnetic fields (0.16 Hz) encouraged release (of what) more successfully than high-frequency fields (1 Hz). By efficiently adjusting release kinetics, magnetomechanical actuation provides new information for intelligent drug delivery and bioscaffold design.

#### 2.2.2. Electro-Adhesion Enhancement

The electro-adhesive behavior of protein–polysaccharide hydrogels arises from the ionizable groups within their molecular structure, or their response to external electric fields. Research in this area remains limited. Upon electrical stimulation, charged moieties such as amino, carboxyl, and sulfonic acid groups undergo oriented migration and rearrangement, facilitating strong electrostatic interactions with oppositely charged components of mucosal tissues [46]. When electrically activated, electro-adhesive hydrogel (e-GLUE) allows for strong and extended mucosal retention. Its soft qualities enable it to conform to the intricate geometry of the gastrointestinal system, and electro-adhesion creates strong cross-links with multi-anionic proteins in the mucosa by activating cationic polymers with an external electric field. By achieving an adhesion energy 30 times higher than traditional materials and effectively tackling the problem of long-term adhesion within the gastrointestinal tract, this dual mechanism allows e-GLUE to maintain stable adhesion within the dynamic environment of bodily fluid immersion and gastrointestinal motility [46].

To accomplish reversible electroadhesion by low-controlled ion migration, Lu et al. [47] created an anionic κ-carrageenan hydrogel and a physiological saline-based nonionic polyacrylamide hydrogel. When an electric field and pH variations work together, this electroadhesion creates a double ion layer at the adhesion contact. The adhesion strength dropped to less than 0.1 kPa when reverse voltage was introduced. Separation resulted from ion migration and diffusion, upsetting the double ion layer. After 1000 cycles, the electro-adhesive hydrogel system’s charge storage capacity changed by less than 7%, setting the stage for the creation of highly effective, electrically controlled adhesive hydrogels. An electric field-induced integrated asymmetric wet-adhesive hydrogel (E-DCb) was created by Xu et al. [48] A gradient charge distribution was created inside the gel by using an electrostatic field to separate cations and anions. As a result, there was notable asymmetry in the submerged adhesion strength, with 97 kPa on the cationic side and only 25 kPa on the anionic side. This hydrogel would have promised applicable prospect in biomedical applications since it can firmly attach to organ wounds while avoiding postoperative tissue adhesions on the opposite side.

In contrast to endogenous stimulus–responsive systems, externally regulated hydrogels allow spatiotemporal control over drug release with high precision and reversibility; nevertheless, their clinical translation remains constrained by the need for auxiliary equipment, limited penetration depth, and challenges in achieving uniform stimulation within the dynamic gastrointestinal environment.

### 2.3. Release of Physical Adsorption

The complex hydrogel known as mucus is made up of water (90–95%) [49], proteins, carbohydrates, lipids (1–2%), salts, antibodies, cells, and cellular detritus [50,51]. Mucin (1–5%) [52] is its main protein component. It can be released or cell-bound, producing a biological hydrogel network and controlling surface chemistry [53,54]. Two separate strata make up the mucosal layers of the stomach and colon: an inner tight adhesion layer that firmly adheres to the epithelium and an outer loose adhesion layer that interacts with luminal contents. Lubrication, particle trapping, and microbial colonization are made easier by the outer layer’s low density, high permeability, and low friction. Maintaining intestinal homeostasis depends on the inner layer’s thick structure, which is crucial for mucosal defense and selective nutrient penetration [55].

The main interaction between protein–polysaccharide hydrogels and the gastrointestinal mucosa is physical adsorption, which mostly involves hydrophobic, hydrogen bonding, and electrostatic forces. Chitosan is a type of polysaccharide that possesses the richest natural positive charge. The protonation of its amino groups under the acidic environments (-NH_3_^+^) permits active electrostatic interactions with negatively charged sialic acid residues on the mucosal surface, which turns to be the most pervasive physical adsorption process. Adhesion is made possible by the protein–polysaccharide hydrogel’s numerous -OH, -NH_2_, and -COOH groups, which can create both covalent and non-covalent interactions with matching groups on the intestinal mucosa. Complexes of collagen, gelatin, and polysaccharides show great potential in improving mucosal adhesion and stomach retention [56]. Through hydrogen bonding and electrostatic interactions, collagen/gelatin that is high in proline and glycine forms stable complexes with polysaccharides, extending the period that food is retained in the stomach and demonstrating superior mucosal adherence [57]. When compared to chitosan alone, Tejada et al. [58] showed that chitosan–gelatin composite formulations have better adhesion and mechanical qualities [59]. An oral hydrogel based on chemically crosslinked silver ear polysaccharide (TP) and 1,4-butanediol diglycidyl ether (BDDE) was created by Wang et al. [60]. Adhesion is improved by the negatively charged hydrogel’s electrostatic attraction to positively charged areas of the inflammatory colonic mucosal surface. The crosslinked hydrogel successfully resisted intestinal peristalsis and fluid washout thanks to its exceptional mechanical strength and adhesive stability. At ulcer locations, it created a strong artificial barrier that shielded the injured mucosa and allowed for long-term local medication release. Additionally, tilapia collagen peptides (TCP) and chitosan worked together to improve wound healing while avoiding microbial inactivation of TCP [61,62]. Through electrostatic interactions, anionic alginate (ALG) and cationic thiolated chitosan (CS-NAC) create a polyelectrolyte complex framework in stomach acid settings. While CS-NAC’s grafted sulfhydryl (-SH) groups engage in disulfide bond exchange reactions with cysteine residues in mucosal proteins to form strong covalent bonds that greatly improve adhesion strength and durability, the protonated amino groups of the compound create strong electrostatic attraction with negatively charged mucosal surfaces [63].

### 2.4. Mechanism for Coordinating Multiple Responses

In complex physiological situations, multiresponsive hydrogels overcome the limitations of single-response systems. By reacting to several inputs at once, including pH, enzymes, and redox states, these systems can precisely control the release of drugs or proteins through chemical and physical changes. This makes multistage, precisely timed administration possible, reducing systemic exposure and adverse effects while guaranteeing effective drug release at target areas. These soft materials have become effective instruments in biological applications such as drug administration because they can react to a variety of external stimuli and frequently work in tandem with pH responses [64]. In hydrogels, pH and enzyme responsiveness are typical dual-response mechanisms. To control medication release, hydrogels that include polysaccharides with carboxyl and amino groups—such as chitosan and hyaluronic acid—swell or compress at varying pH levels due to charge shifts. For example, hydrogels swell to release medications in tumors and inflammatory areas under acidic conditions (pH < 6.5). Simultaneously, protein chains or complex networks can be modified to incorporate enzyme-sensitive peptide sequences that react to certain enzymes (like MMPs) by causing breakdown and releasing the payload [65]. As shown in Table 1, by integrating multiple endogenous and exogenous stimuli, multi-responsive hydrogels overcome the inherent limitations of single-response systems, achieving improved targeting accuracy, release controllability, and environmental adaptability; nevertheless, the increased material complexity and manufacturing challenges necessitate careful balance between functional integration and translational feasibility.

## 3. Applications and Prospects of Polysaccharide–Protein Hydrogels

### 3.1. High Encapsulation Capacity and Stability (Using Curcumin as an Example)

Curcumin, a natural yellow pigment derived from turmeric, has high antioxidant and anti-inflammatory properties that support digestive health, decrease inflammation, and have anticancer potential [73]. However, its low bioavailability is caused by its limited gastrointestinal absorption, quick metabolism, low light stability, and poor water solubility [74]. Hydrogels and other carriers are therefore frequently used to improve their stability and distribution. For instance, Amirhossein et al. [75] used whey protein isolate (WPI) and Arabic xylan (AX) that was separated from sesame husk waste to create a thermally induced composite hydrogel encapsulating curcumin. This hydrogel has outstanding heat stability, great elasticity, and good water retention (>99%). It can successfully encapsulate curcumin via hydrogen bonding and hydrophobic interactions with an encapsulation rate of 98.66%. It can also enhance curcumin’s antioxidant activity and enable controlled release under simulated gastrointestinal conditions. Glucono-δ-lactone (GDL) was used by Li et al. [76] to create a curcumin hydrogel through hydrophobic and electrostatic interactions between casein and black fungal polysaccharides. This method greatly improved the stability of curcumin administration and increased encapsulation efficiency by 64% as compared to single-polysaccharide hydrogels. In addition, Bu et al. [77] encapsulated curcumin using a composite hydrogel (THIH) made of hawthorn pectin (HP) and tenebrio molitor protein (TMP) at pH 3.35, a 2:1 mass ratio, and 15 mmol/L Ca2+. This gel exhibits a dense structure with a small pore size, demonstrating excellent rheological properties and water retention capacity. It significantly enhances curcumin’s storage stability, sustained-release performance, and antioxidant activity, exhibiting favorable controlled-release effects in simulated gastrointestinal environments. It represents a promising delivery system for active ingredients.

### 3.2. Targeted Delivery and Intelligent Controlled-Release (EGCG as an Example)

EGCG has exceptional antioxidant characteristics and is the most potent active ingredient in green tea [78,79] that has a significant role to play in osteogenesis, anticancer effects, cardioprotection, anti-obesity, and neuroprotection. It can also enhance the microecological environment and encourage the development of advantageous gut bacteria at low concentrations as a crucial prebiotic [80]. However, in vivo, EGCG has limited absorption rate and low stability. It has poor membrane permeability, limited stability in alkaline environments, and susceptibility to oxidative hydrolysis and inactivation [81]. To improve stability and bioavailability, polysaccharide–protein hydrogel encapsulation and distribution is a helpful strategy. By combining improved hydrogen bonding networks via tannic acid-modified nano-hydroxyapatite (TA@n-HA) with electrostatic interactions between carboxymethyl konjac glucomannan (CKGM) and gelatin (G), Wang et al. [82] were able to accomplish pH-sensitive regulated release of EGCG. This technology is appropriate for intestine-targeted distribution because of its high encapsulation efficiency, superior thermal stability, and regulated degradation. Lin et al. [83] used pea protein–alginate dual-gel beads to co-encapsulate and release hydrophilic and hydrophobic active substances in a regulated manner. Both of them showed good swallowability and intestinal-targeted release effects by controlling structural strength and release behavior using ionic crosslinking, which makes it appropriate for nutritional delivery to people who have difficulty swallowing.

### 3.3. Safeguarding Highly Sensitive Substances (Using Probiotics as an Example)

Probiotics are living bacteria that promote intestinal health when taken in the right proportions. For decades, they have attracted a wide interest because of their potential to improve immunity, promote nutrient absorption, lower the risk of allergies, alleviate gastrointestinal diseases, constipation, diarrhea, irritable bowel syndrome, and maintain gut microbiota balance [84]. Probiotics’ capacity to colonize the gut can be severely diminished by physiological variables, including bile salts in the intestine and low stomach pH, as well as storage conditions. A prebiotic hydrogel based on guar gum (GG) and flaxseed arabic xylan (AX) was created by Mehta et al. [85]. The main function of this hydrogel is to physically enclose bacterial cells in a network of polysaccharides, creating a stable three-dimensional structure by hydrogen bonding. This design allows for significant release in intestinal fluid (about 78%) and minimal release in stomach fluid (preserving the germs). Liu et al. [86] used TGase-induced crosslinking in conjunction with ultrasonication to create a composite hydrogel of high-molecular-weight citrus pectin (HMP) and soy protein isolate (SPI). Through swelling brought on by an elevated pH, this gel successfully shielded probiotics (Lactobacillus plantarum) from acid and enzymatic breakdown in stomach fluid, allowing for their targeted release in intestinal fluid. Probiotic survival rates were greatly increased by gels that were sonicated for 20 min under UV irradiation and simulated gastrointestinal digestion, as they demonstrated excellent gel strength, water retention, and microstructure. Ma et al. [87] co-encapsulated Lactobacillus plantarum and EGCG in whey protein–polysaccharide microcapsules using electrospray technology. The sturdy structure created by the synergistic interaction of proteins and polysaccharides (such as β-cyclodextrin) improved the microcapsules’ gastrointestinal tolerance, antioxidant capacity, and thermal stability. Together, EGCG and polysaccharides increased bacterial survival rates and markedly increased antioxidant activity, offering an effective defense mechanism for probiotic delivery.

## 4. Challenges and Prospects for Polysaccharide–Protein Hydrogel Development

Before discussing specific technical challenges, it is important to clarify the experimental contexts in which current polysaccharide–protein composite hydrogel systems have been evaluated. The majority of studies summarized in this review rely primarily on in vitro models, including simulated gastric and intestinal fluids, pH-dependent swelling and degradation assays, enzymatic digestion systems, and controlled release experiments, which are widely adopted to elucidate structure–function relationships of gastrointestinal delivery systems [88,89]. These models are highly valuable for mechanistic investigation; however, they cannot fully replicate the dynamic physiological complexity of the gastrointestinal tract, such as peristalsis, mucus turnover, and interindividual variability.

A more limited number of investigations have extended these evaluations to in vivo settings. For example, polysaccharide–protein hydrogels have been demonstrated to significantly enhance the bioavailability and gut retention of encapsulated probiotics compared with their free form in rodent models [90], and double-layer polysaccharide hydrogel carriers have shown markedly improved intestinal colonization and survival of probiotics in animal experiments [91]. These studies typically benchmark composite hydrogels against free drug/probiotic administration or non-encapsulated formulations to assess comparative efficacy. Nevertheless, direct cross-study comparison remains challenging due to differences in disease models, dosing regimens, and evaluation endpoints. This imbalance between extensive in vitro validation and relatively limited in vivo evidence underpins many of the technical and translational challenges discussed below.

### 4.1. Current Technical Challenges

#### 4.1.1. The Balance Between Material Stability and Bioavailability

Maintaining structural integrity in the hostile environment of the digestive system while effectively releasing of active ingredients is the key challenge for polysaccharide–protein composite hydrogels during the gastrointestinal delivery. Traditional hydrogels are vulnerable to structural disintegration because of protein denaturation and excessive protonation of certain polysaccharides in the stomach’s low pH range (pH 1.5–3.0) and mechanical digestion (such as peristalsis). Effective intestinal targeting is limited by insufficient protection of encapsulated probiotics and rapid gel disintegration, leading to premature and excessive release in non-target regions such as the stomach [92,93]. Additionally, the continuous renewal and shedding of the intestinal mucus layer, which acts as a natural barrier, markedly reduces hydrogel residence time. According to experimental data, traditional polysaccharide–protein nanoparticles only stay in the small intestine for 60–88 min [94], which is insufficient for long-term chronic illness therapy.

#### 4.1.2. Issues with Controlled Release and Accurate Targeting

One of the key goals of designing polysaccharide–protein hydrogels is to achieve colon-specific release. However, the targeting accuracy of current methods is still an issue. Strong mucosal adhesion capabilities are found in many natural polysaccharides, including chitosan, which promote the retention of the hydrogel system in intestinal regions but may also cause significant drug sequestration in non-target healthy mucosal sites, thereby limiting effective delivery to lesion areas. Additionally, many receptor-mediated targeting systems depend on identifying receptors that are overexpressed in sick tissues (such as the hyaluronic acid-binding CD44 receptor on tumor cells [95]). However, some normal cells also produce these receptors, which might lead to off-target consequences. One targeting strategy is not sufficiently robust in the complicated vivo environment. For instance, pH-responsive systems offer excellent controllability for oral drug delivery targeting tumors or the colon. However, their clinical application is limited by the complexity of the human physiological environment. Normal and malignant tissues exhibit distinct pH values, with tumor microenvironments typically ranging from 5.8 to 7.2 [96]. Moreover, local pH variations may arise from inflammation, trauma, physical activity, or temperature fluctuations [97]. Consequently, interpatient differences or microenvironmental heterogeneity within a single lesion may lead to premature, delayed, or incomplete drug release from hydrogels designed to respond at pH 7.0, thereby compromising therapeutic efficacy.

#### 4.1.3. Barriers to Scalable Production and Clinical Translation

There are two significant obstacles to the clinical translation of polysaccharide–protein composite hydrogels: they are scalable in production and quality control. Notably, batch variability in natural polysaccharide and protein sources, as well as extraction and purification processes, can compromise quality control [98]. Additionally, there are yet no globally recognized quality assessment methods or green metrics for hydrogels that are organically generated and have complicated polysaccharide–protein compositions [99]. Moreover, the accurate identification of crosslinker residues, such as those from EDC/NHS crosslinking systems, is a significant regulatory hurdle. Furthermore, advanced evaluation methods are needed to elucidate the long-term in vivo fate of implanted hydrogels, including the potential immunological effects of their degradation products, such as modulation of macrophage polarization toward M1/M2 phenotypes [98,100]. Although the number of fundamental research papers on hydrogels has increased rapidly, the rate of clinical technology translation is modest (estimated at less than 20%) [101]. Therefore, to comprehensively address these translational barriers, strong interdisciplinary collaboration is still necessary when expanding laboratory research into larger clinical applications.

### 4.2. Strategies and Directions for Future Development

#### 4.2.1. Intelligent Response

Research on multi-stimuli-responsive systems has gained prominence as a strategy to improve targeting accuracy. As mentioned previously, the design of next-generation hydrogels is shifting from single pH responsiveness to synergistic pH/enzyme/redox multi-response mechanisms. For example, Tang et al.’s chitosan/HP-β-CD hydrogel system shows dual sensitivity to enzymes and pH. In addition to dissociating in the colon’s acidic milieu, it also accomplishes “dual-stage targeting” by targeting macrophages, which significantly increases drug accumulation in inflammatory areas [102]. In ulcerative colitis models, this advanced design enables precise co-delivery of kaempferol- and rhubarb-derived nanovesicles, exhibiting greater efficacy than conventional drugs. Therefore, the integration of multiple stimuli-response mechanisms represents a key direction for enhancing the precision and adaptability of next-generation gastrointestinal delivery platforms.

#### 4.2.2. Functionalization of Materials

Biomaterials innovation is another key strategy for enhancing responsive performance. Dynamic hydrogels composed of dual polysaccharides (CCS/CMC-Na) and Ti_3_C_2_ MXene represent a significant advancement in responsive biomaterials. The incorporation of MXene nanosheets imparts broad-spectrum ROS-scavenging activity and enhanced mechanical strength, markedly reducing disease severity in DSS-induced colitis models and outperforming the first-line drug 5-ASA. This system also simplifies administration by eliminating dual-channel dosing and maintains excellent biocompatibility for over 50 days [10]. Additionally, integrating therapeutic agents directly into the delivery matrix to form “self-healing systems” offers an effective strategy to overcome the limitations of single-function carriers. Indian researchers developed a psyllium gum–gum arabic/polyacrylamide hydrogel incorporating natural antibacterial and antioxidant components. It exhibited a DPPH radical scavenging activity of 14.1 ± 0.7% and strong inhibitory effects against *Staphylococcus aureus* and *Escherichia coli* when combined with the antibiotic meropenem [103]. The therapeutic potential of individual materials is significantly enhanced by this “multi-functional material” designing concept. Overall, material functionalization enables hydrogels to integrate protection, targeting, and bioactivity modulation within a single delivery platform, thereby enhancing therapeutic precision in complex gastrointestinal environments.

#### 4.2.3. Precision in Preparation

The translation of multifunctional hydrogels from conceptual design to practical application increasingly relies on the advancement of manufacturing technology. Recent progress in 3D bioprinting has provided new opportunities for fabricating complex hydrogen-based structures with high spatial precisions. For instance, Chu et al. reported a whey protein microgel-based “bioink” that enables the printing of intricate biological architectures such as aortic valves and human ears, with resolutions of up to 200 μm. The bioink’s remarkable shear thinning and rapid self-healing properties ensured both smooth printing and structural stability [21], suggesting potential applications in gastrointestinal disorders. Looking forward, the integration of modern manufacturing techniques such as microfluidics with intelligent material standardization is expected to enable more accurate and reproducible hydrogel production. Moreover, the incorporation of artificial intelligence, particularly AI-assisted design, is expected to further accelerate the discovery and optimization of advanced hydrogel systems for biomedical applications. In conclusion, advancements in meticulous preparation techniques are vital for attaining consistent hydrogel structures and adjustable performance, which are necessary conditions for large-scale production and clinical application.

### 4.3. Polysaccharide–Protein Composite Hydrogels’ Safety by Design and Clinical Translations

From an industrial and translational standpoint, the successful transition of polymeric hydrogel products from lab research to clinical application is largely dependent on regulatory approval and standardized manufacturing systems [104]. Only a small number of traditional, non-responsive hydrogels have so far successfully finished regulatory pathways and entered clinical use [105]. Developing new hydrogel-based delivery systems usually involves significant financial investment and extended evaluation periods. Material safety and biocompatibility, adherence to Good Manufacturing Practice (GMP) to guarantee sterility and batch-to-batch consistency, and clear regulatory classification—such as designation as medical devices or drug-device combination products—are typically important regulatory considerations. Each of these categories has specific approval requirements under various regulatory frameworks [106].

In this regard, a quality-by-design (QbD) framework offers a logical approach to enhancing the translational viability of hydrogels made of polysaccharide and protein for targeted delivery in the gastrointestinal tract. Product performance, safety, and manufacturing robustness are all influenced by critical quality attributes (CQAs), critical material attributes (CMAs), and critical process parameters (CPPs), all of which are systematically identified and controlled by QbD. CPPs control the reproducibility and robustness of the hydrogel manufacturing process, CMAs explain the inherent physicochemical properties of raw materials that affect these properties, and CQAs specify the fundamental qualities needed to guarantee therapeutic efficacy and safety. CQAs, CMAs, and CPPs are intricately linked, and their integrated management is essential for the effective performance of polysaccharide–protein composite hydrogels. Employing QbD principles in the design and production of hydrogels is anticipated to enhance consistency and expedite the approval process for gastrointestinal delivery systems in clinical applications.

From a translational and quality-by-design (QbD) viewpoint, preformulation studies represent a vital intermediary phase connecting material design and the clinical advancement of polysaccharide–protein composite hydrogels for gastrointestinal administration. Before optimizing formulations and scaling up, a thorough physicochemical characterization of raw materials is necessary, encompassing molecular weight distribution, charge density, functional group availability, and interaction affinity between polysaccharides and proteins. In parallel, hydrogel formation behavior, crosslinking efficiency, and network homogeneity should be systematically evaluated, as these factors directly influence mechanical integrity and stimulus responsiveness.

Furthermore, preformulation assessments under simulated gastrointestinal conditions—such as swelling, degradation, and mechanical stability in gastric and intestinal environments—are essential for predicting in vivo performance and minimizing the risk of premature structural failure. Compatibility studies between hydrogels and encapsulated bioactive agents, together with preliminary release profiling and mucoadhesion evaluation, provide early insights into therapeutic feasibility and targeting efficiency. Collectively, these preformulation investigations establish a rational foundation for defining CQAs, optimizing CMAs and CPPs, and facilitating reproducible manufacturing and regulatory evaluation of polysaccharide–protein composite hydrogels.

## 5. Summary and Prospects

Polysaccharide–protein composite hydrogels have emerged as promising vehicles for targeted gastrointestinal delivery due to their enhanced biocompatibility, degradability, and environmental responsiveness. Their primary benefits include safeguarding encapsulated drugs or probiotics from harsh gastrointestinal environments and enabling targeted release through pH- or enzyme-responsive mechanisms, thereby enhancing oral bioavailability for diverse therapeutic agents. Nonetheless, obstacles such as inadequate mechanical durability, the potential for premature gastric rupture, and the necessity to reconcile targeting accuracy with streamlined fabrication persist as significant impediments to their wider utilization.

Despite these challenges, future advancements in polysaccharide–protein composite hydrogels for targeted gastrointestinal delivery will likely emphasize the strategic amalgamation of material design and functional responsiveness. Exact chemical modification, hybridization techniques, and sophisticated crosslinking methods will be pivotal in customizing mechanical properties, degradation characteristics, and stimulus responsiveness in intricate gastrointestinal environments. Multifunctional systems that integrate pH sensitivity, enzyme-mediated degradation, and dynamic network interactions are expected to surpass single-response hydrogels by offering improved spatiotemporal regulation of payload protection and release.

In addition to material innovation, effective clinical translation will increasingly rely on scalability, standardization, and regulatory compliance. The development of strong manufacturing processes, predictive preclinical evaluation models, and quality-by-design optimization frameworks will be essential for ensuring reproducibility and regulatory compliance. Furthermore, innovative tools like advanced manufacturing methods and data-driven material design are anticipated to expedite the optimization of hydrogel systems for patient-relevant performance. These efforts may enable the transition of polysaccharide–protein composite hydrogels from laboratory prototypes to clinically viable gastrointestinal delivery systems, connecting advanced material functionality with practical therapeutic application.

## Figures and Tables

**Table 1:** Release Mechanisms and Applications for Different Response Types.

Number	Response Mechanism Type	Carrier/ Material	Conditions and Release Mechanism	Application/ Drug	Reference
1	pH/Enzyme	HEMA-MAA copolymer, DMAAB (azobenzene-type) crosslinking agent	Low swelling in gastric acid (pH 1.2) limits release; MAA ionization promotes swelling in colonic neutral environment (pH 7.4); Colonic microbial azoreductase degrades azo bonds, accelerating release.	Poorly water-soluble drugs (such as mesalazine)	[66]
2	pH/Enzyme	Dopamine-modified sodium alginate (DA-SA), konjac glucomannan (KGM), and chitosan (CS) outer coating	In gastric acid (pH 1.2), CS/SA contracts and densifies, restricting release; in the neutral intestinal environment (pH 6.8–7.4), the gel swells; in the colon, β-mannanase degrades KGM, disrupting the structure to achieve targeted release; DA enhances mucosal adhesion.	Mesalazine (Ulcerative Colitis)	[67]
3	pH/Redox	Sodium Alginate (SA) and Dialdehyde Starch (DAS) Composite Hydrogel Beads	Carboxyl group dissociation increases swelling and facilitates release in alkaline settings, whereas disulfide bond breakage upsets the network structure under reducing situations (such as in the presence of glutathione (GSH).	Pesticide Sustained-Release	[68]
4	Redox/NIR Light	Gold nanorods (AuNRs) with glutathione-extended polyurethane urea electrospun membranes made of poly (CEGS)	Disulfide bond breakage facilitates drug release under reducing circumstances; AuNRs provide a photothermal effect (heating up to 42.5 °C) upon exposure to near-infrared light (810 nm), which further initiates drug release to achieve synergistic chemotherapy-photothermal treatment.	Paclitaxel (PTX) (Synergistic Chemotherapy-Photothermal Therapy)	[69]
5	pH/ROS	SA-SPBA	Borate bonds break to allow for intelligent drug release in the acidic environment of wounds (pH 5.6) and high ROS concentrations; at the same time, the hydrogel scavenges excess ROS, lowers inflammation, and speeds up healing.	Probiotic (Lactobacillus rhamnosus) (Antibacterial and Wound-Healing)	[70]
6	Magnetic/pH	Twin-Dome Microbots (TDMs) with Calcium Alginate Shells and Magnetic Chitosan Microsphere (mCS) Cores	The shell remains stable in the acidic gastric environment to protect the drug-loaded microspheres, dissolves in the alkaline intestinal environment for sustained drug release, and enables precise navigation under an external magnetic field.	Intestinal-targeted sustained-release (e.g., DOX)	[71]
7	pH/Enzyme	TEMPO-oxidized cellulose nanofiber (TOCNF) carrier, ZIF-8 nanoparticles, and pectin protective layer multi-responsive hydrogel system	To accomplish pH-responsive release, ZIF-8 breaks down in acidic settings; to achieve enzyme-responsive release, microorganisms produce pectinase, which breaks down pectin. Intelligent, on-demand, and effective release is made possible by the synergistic action of two stimuli.	Drug-loading molecule (carvacrol)	[72]

## Data Availability

No new data were created or analyzed in this study.
